# Eliciting human intelligence: police source handlers’ perceptions and experiences of rapport during covert human intelligence sources (CHIS) interactions

**DOI:** 10.1080/13218719.2020.1734978

**Published:** 2020-05-06

**Authors:** Jordan Nunan, Ian Stanier, Rebecca Milne, Andrea Shawyer, Dave Walsh

**Affiliations:** a Institute of Criminal Justice Studies, University of Portsmouth, Portsmouth, UK; b Liverpool John Moores University, Liverpool, UK; c School of Law, De Montfort University, Leicester, UK

**Keywords:** covert human intelligence source, covert policing, human intelligence, informants, police perceptions, rapport

## Abstract

Rapport is an integral part of interviewing, viewed as fundamental to the success of intelligence elicitation. One collection capability is human intelligence (HUMINT), the discipline charged with eliciting intelligence through interactions with human sources, such as covert human intelligence sources (CHIS). To date, research has yet to explore the perceptions and experiences of intelligence operatives responsible for gathering HUMINT within England and Wales. The present study consisted of structured interviews with police source handlers (N = 24). Rapport was perceived as essential, especially for maximising the opportunity for intelligence elicitation. Participants provided a range of rapport strategies while highlighting the importance of establishing, and maintaining, rapport. The majority of participants believed rapport could be trained to some degree. Thus, rapport was not viewed exclusively as a natural skill. However, participants commonly perceived some natural attributes are required to build rapport that can be refined and developed through training and experience.

## Introduction

In security contexts, the collection of intelligence is deemed critical to both proactive and reactive forms of investigation (Innes & Sheptycki, 2004; James, 2013). A variety of methods are available to agencies, both overt and covert, in order to collect intelligence (Chappell, 2015). One collection capability is human intelligence (HUMINT), the discipline charged with eliciting intelligence through interactions with human sources, such as covert human intelligence sources (CHIS). CHIS play a significant role within HUMINT (James, Phythian, Wadie, & Richards, 2016) and are defined in England and Wales within Section 26(8) of the Regulation of Investigatory Powers Act 2000 (RIPA). For the purposes of RIPA, a person should be considered to be a CHIS when:

- He establishes or maintains a personal or other relationship with a person for the covert purpose of facilitating the doing of anything falling within paragraph b or c;
- He covertly uses such a relationship to obtain information or to provide access to any information to another person; or
- He covertly discloses information obtained by the use of such a relationship, or as a consequence of the existence of such a relationship.

Within England and Wales, law enforcement CHIS are managed within dedicated source units and interact with police officers known as source handlers. While current training focuses primarily on *tradecraft* including counter-surveillance measures, to maximise intelligence elicitation from a CHIS, the present research holds that there are available tools and techniques that may assist source handlers and CHIS intelligence interactions through an appreciation and application of psychological science.

### Interviewing for intelligence

For the purposes of this article, the term *interviewing* is used in its broadest sense to include an intelligence interaction between a police source handler (the *interviewer*) and a CHIS who may have information of interest (the *interviewee*). The elicitation of intelligence (i.e. an intelligence interview) can be broken down into three key sections (Stanier & Nunan, 2018). First is the use of rapport to try and secure the interviewee’s engagement, to assist with recruiting the interviewee as a CHIS and to maintain the longevity of an elicitation relationship. Second, with engagement obtained, the interviewer’s role is to elicit detailed and reliable information through appropriate interviewing techniques. The third and final stage is to assess the integrity of the information obtained, which is undertaken through a process of assurance, corroboration and validation, all which make up part of what is known as the provenance (Stanier, 2013).

The majority of intelligence interviews should strive to elicit the most detailed and reliable accounts from an interviewee, which can provide an insight into the workings of individuals and groups of individuals regarding past and future events (Chappell, 2015). Detailed and reliable information is essential because it helps inform subsequent investigative decision-making (James, 2013). It is crucial though that intelligence should not be obtained at any cost (Alison & Alison, 2017; Intelligence & Security Committee of Parliament, 2018). Interviewing must be ethically conducted in order to obtain intelligence that is legally admissible and factually reliable (Alison & Alison, 2017). As research has found, the history of police interviewing in England and Wales is chronicled with the many consequences of unethical and ineffective interviewing practices (e.g. Poyser, Nurse, & Milne, 2018). Hence, a reliance on the existing evidence-base concerning the psychology of interviewing should counter policing practices based on anecdotal experiences. The focus of this article concerns the first key section of an intelligence interview, namely rapport, as rapport is also understood to be a working alliance (Billingsley, 2003; Kleinman, 2006; Tickle-Degnen, 2002; Vanderhallen, Vervaeke, & Holmberg, 2011).

### Rapport: cultivating HUMINT

Operational circumstances vary, and many opportunities to gather HUMINT occur within a collapsing time frame, for example:

- Conducting an *exploratory prison debrief* to elicit information from a prisoner within the 45-min England and Wales prison legal visit period;
- During a *port stop*, whereby a passenger arrives into England and Wales, and either passes through passport control or collects baggage transfers to another journey;
- A *cold call pitch* in person or via telephone to a person of interest to assess their willingness to meet source handlers at a later date; and
- Within the *police custody block*, where prisoners are detained and regulated by the Police and Criminal Evidence Act 1984.

Hence, the cultivation of potential new sources of intelligence relies heavily upon the application of effective rapid rapport-building techniques, such as identifying the *hooks* (a way to gain attention and build rapport, e.g. personal interests, lifestyle characteristics or motivations) of an individual to influence cooperation (see Cooper, 2011). Within the context of HUMINT, rapport can be defined as ‘developing and maintaining a working relationship with a human source, by managing their motivations and welfare, whilst ensuring they understand the purpose of the relationship in order to secure reliable intelligence’ (Stanier & Nunan, 2018, p. 232). Alongside this definition, the concept of *operational accord* (Kleinman, 2006) acknowledges that an interviewer–interviewee relationship needs mutual affinity and conformity, thus requiring the interviewer to appreciate the interviewee’s concerns and intentions and the desired outcomes of the interaction (Evans, Meissner, Brandon, Russano, & Kleinman, 2010). Tickle-Degnen and Rosenthal (1990) stress the importance of building and then maintaining rapport, highlighting three interrelating elements: *mutual attentiveness*, *positivity* and *coordination*. Within early interactions (i.e. building rapport), emphasis is placed on *mutual attentiveness* and *positivity*, with *mutual attentiveness* and *coordination* considered more important in subsequent interactions (i.e. maintaining rapport). Thus, once rapport has been established, it is important to maintain that relationship over time in order to cultivate HUMINT, especially in relation to CHIS.

Overly officious introductions have been found to generate negative perceptions from interviewees, especially when this incorporates a lack of rapport and a warning to the interviewee about lying (MacDonald, Keeping, Snook, & Luther, 2016), whereas positive interviewee perceptions have found to be formed when rapport is applied successfully within the first few minutes of an interaction (Zunin & Zunin, 1972). Furthermore, throughout the interaction, an overly formalised delivery aligned with functional pre-determined questions has been shown to impede rapport (Milne & Bull, 1999). Thus, the use of nonverbal techniques (e.g. mirroring behaviour and displaying understanding via empathy, especially when eliciting highly personal information) and verbal techniques (e.g. establishing a common ground) has been reported by interviewers as effective rapport-building techniques (Abbe & Brandon, 2013; Vallano, Evans, Schreiber Compo, & Kieckhaefer, 2015). Nonetheless, while establishing rapport may be sufficient to influence the overall quality of the interaction, it is also argued that maintaining rapport throughout the interaction is crucial (Abbe & Brandon, 2013; Leach, 2005; Walsh & Bull, 2012). Thus, effective techniques that build and then maintain rapport help exercise ‘social influence, and educing information from a source’ (Abbe & Brandon, 2013, p. 237).

### Rapport-based interviewing

While the short operational window offered by some of the previously noted scenarios means that the interviewer is required to deploy rapid rapport-building techniques, other circumstances, such as a remanded/sentenced prisoner or an existing CHIS relationship, allow for a more patient, measured and long-term approach. Rapport is viewed by practitioners both as an important part of the interview process and as being fundamental to the success of information and intelligence elicitation (Russano, Narchet, Kleinman, & Meissner, 2014; Semel, 2012). In fact, rapport is considered important across numerous interviewing contexts. For example, rapport forms a key role in England and Wales’ PEACE model of investigative interviewing (an acronym for the five phases of the interview process; Planning and preparation; Engage and explain; Account; Closure; and Evaluation). PEACE is underpinned by the Police and Criminal Evidence Act 1984, and helped to shift the focus of interviewing in England and Wales from accusatory and confession-driven methods to that of information gathering (Clarke & Milne, 2001, 2016; Moston & Engelberg, 2011).

Rapport is often likened to friendship (Clark, 2014), a common theme reported across numerous interviewing professionals. For example, Russano et al. (2014) interviewed experienced military and intelligence interrogators, revealing that they believed noncoercive approaches to be superior to coercive approaches. Additionally, rapport has been shown to assist with securing disclosure from high-value detainees, which are deemed vital sources of information to identify emerging threats and disrupt terrorist planning (Goodman-Delahunty, Martschuk, & Dhami, 2014). Goodman-Delahunty et al. (2014) found that when rapport (i.e. noncoercive strategies) was employed in these particular contexts, information was more likely to be disclosed and disclosed in more detail, and was done so earlier within the interview.

Redlich, Kelly, and Miller (2014) examined U.S. military and federal interrogators’ perceived effectiveness and frequency of using various interrogation techniques. Rapport- and relationship-building techniques were perceived as the most effective strategies, regardless of the intended outcome and context of the interrogation, and, more importantly, rapport- and relationship-building techniques were used most often, especially when compared to confrontational techniques (Redlich et al., 2014). Moreover, Goodman-Delahunty and Howes (2016) interviewed intelligence and investigative interviewers from Asian-Pacific jurisdictions, regarding their rapport-building techniques utilised with high-value interviewees. These interviews were analysed in line with the principles of persuasion outlined by Cialdini (1993), with liking and reciprocity discussed as the most frequently reported rapport-building strategies (Goodman-Delahunty & Howes, 2016).

In addition to influencing disclosure, research has explored the use of rapport and its influence on memory recall. Rapport building has been shown to enhance the accuracy of interviewee recall and ‘diagnosticity of evidence obtained from suspects’, by reducing the amount of inaccurate and misinformation reported (Vallano et al., 2015, p. 369) – for example, by personalising the interview and transferring the control of the recall process to the interviewee, which is likely to reduce the interviewee’s anxiety, creating an environment that can maximise recall (Memon, Wark, Holley, Bull, & Koehnken, 1997). The positive motivational stance of such rapport-based interviews may encourage the interviewee to try harder and attempt multiple memory recalls (Memon et al., 1997), which, together with the use of open-ended questions, should maximise the elicitation opportunity (Vallano & Schreiber Compo, 2011).

Although rapport is considered important to interviewing and gathering information, limited research has investigated real operational field data to carefully and systematically define the behaviours that underpin rapport. Therefore, while professionals believe rapport works and self-report that they use it, this is insufficient evidence that rapport actually works. However, recent research has revealed that rapport exists within contemporary police interviews and that it is important in order to obtain information (Bull, 2014). Nevertheless, Walsh and Bull’s (2012) investigation of real-world police interviews with fraud suspects identified that opportunities to establish rapport were often missed, and even when rapport was established, it was infrequently maintained. Interestingly, a satisfactory outcome was five times more likely when interviewers managed to establish and maintain rapport throughout (Walsh & Bull, 2012).

A key development within the rapport literature was the creation of a rapport coding framework that could be applied within an operational setting, known as ORBIT (Observing Rapport-Based Interpersonal Techniques; Alison, Alison, Noone, Elntib, & Christiansen, 2013). ORBIT was developed from the counselling literature and is founded on well-researched methods of observing interpersonal skills (Tickle-Degnen & Rosenthal, 1990), particularly motivational interviewing (Miller, Moyers, Ernst, & Amrhein, 2008; Miller & Rollnick, 1992) and the interpersonal behaviour circle (Birtchnell, 2014; Freedman, Leary, Ossorio, & Coffey, 1951; Leary, 1957). ORBIT’s framework measures rapport through empathy, empowerment, respectfulness and open-mindedness (e.g. motivational interviewing; Alison, Giles, & McGuire, 2015; Rollnick & Miller, 1995) and interpersonal behaviours coded as either adaptive (beneficial to communication) or maladaptive (impedes communication; Alison et al., 2015; Birtchnell, 2014) in relation to intelligence yield (Alison & Alison, 2017).

Alison et al. (2013) utilised the ORBIT framework to analyse audio and video footage of terrorist interrogations from 181 convicted suspects. Building rapport was identified as important in securing information disclosures from terrorists as it was positively associated with adaptive behaviours of communication, which consequently increased intelligence yield (Alison et al., 2013). Similar results were found by Alison et al. (2014), whereby an adaptive rapport-based interrogation style (e.g. the use of respect, dignity and integrity) was found to be an effective approach for reducing suspects’ use of counter-interrogation tactics (e.g. no comment interviews, retraction of statements or claiming lack of memory).

Additionally, Christiansen, Alison, and Alison (2018) examined the interpersonal behaviours of police interviewers across interviews with convicted terrorist suspects in a naturalistically occurring environment. Using ORBIT, their results demonstrated that maladaptive behaviours were associated with the suspect shutting down, while adaptive passive behaviours (e.g. humble and seeks guidance) were effective in the first interview. This tactic did not produce the same effects, however, in the final interview, where cooperative adaptive interviewing behaviours (e.g. respect and trust) were associated with improved adaptive detainee behaviours throughout, highlighting the importance of being flexible in adopting different interviewing styles over the course of numerous interactions. Interestingly, such findings seem applicable to the CHIS relationship, whereby numerous interactions occur over a period of time, ultimately aiming to collect intelligence.

Despite the importance of rapport (as highlighted in the literature), no research has addressed the topic of rapport between source handlers and CHIS. As a consequence of this research gap, the present study aimed to develop our understanding of rapport with a neglected sample of police officers (i.e. source handlers). Hence this researched explored source handlers’ (a) perceptions, experiences and definitions of rapport in contrast to previous research, and (b) perceptions regarding whether rapport can be trained and, if so, what methods are suggested to enhance rapport practices. The present study forms part of a wider ongoing programme of research, by conducting structured interviews with police source handlers concerning their perceptions and experiences in relation to gathering intelligence from human sources.

## Method

### Participants

Participants consisted of 24 police source handlers (96% male; 4% female) from several counter-terrorism dedicated source units across England and Wales. The mean age of participants was 44 years (range = 33−59 years), with a mean time spent as a source handler being 6 years (range = 1−15 years).

### Materials

The current research tailored Goodman-Delahunty and Howes’ (2016) rapport interview questions for the context of police source handler interactions with CHIS. Responses from eight questions that were within a longer structured interview protocol (*N* = 32) are discussed within this article, all of which concern the topic of rapport.

### Procedure

Individual gatekeepers were established from each counter-terrorism dedicated source units by the second author, which provided access to a unique sample of police officers. A purposive sampling method was then employed, as the specific criteria required for participants to be eligible for this research were being a police officer (a) who worked in a counter-terrorism dedicated source units and (b) who interacted with CHIS. Having obtained ethical authorisation from the first author’s university and CREST (Centre for Research and Evidence on Security Threats), structured interviews were conducted by the first author with participants who met the inclusion criteria. Spoken interviews (*n* = 15) lasted between 19 and 55 min (*M* = 37 min), which were audio recorded for later transcription and data analysis. The protection of the participants’ identities was of utmost importance due to the sensitive nature of their work. Hence, alternative methods were put in place; those interviewed face-to-face (*n* = 11) had the option to either sign the consent form or provide consent verbally on the audio recording device to refrain from providing a written name/signature. Due to the operational commitments and availability of the participants, some participants provided their responses via an audio recorded internet/phone interview (*n* = 4) or by written responses via the designated gatekeeper’s email (*n* = 9). In addition, a condition of participation included that participants would read through the transcript of their interview and provide approval for their transcript to be used for the current study.

### Data analysis

A systematic, thematic analysis was undertaken, which followed the principles outlined by Braun and Clarke (2006, 2012). Thematic analysis is a flexible and accessible qualitative method that allows the author to view and develop an understanding of shared perceptions and experiences (Braun & Clarke, 2012). In line with the thematic analysis principles, this research progressed in three stages. First, the overall research question was developed: how do source handlers perceive and experience rapport with CHIS? Second, in order to address the overarching research question, the interviews asked the following questions to source handlers:

1. How would you define rapport within the context of an intelligence gathering interview/debrief?
2. What is the importance of rapport in an intelligence gathering interview/debrief?
3. What strategies for *establishing* rapport do you find to be *most* effective?
4. What strategies for *establishing* rapport do you find to be *least* effective?
5. What strategies for *maintaining* rapport over the relationship with a source do you find to be *most* effective?
6. What strategies for *maintaining* rapport over the relationship with a source do you find to be *least* effective?
7. How do you know when rapport has been achieved (or not) with a source (i.e. what evidence or indicators do you look for)?
8. Do you think that rapport can be trained?
  - If yes, what aspects?

Third, the data analysis stage was partly informed by the authors’ previous knowledge of the rapport literature, as well as the discussion points raised by participants whilst coding the data. For example, prior to data collection, the first author was aware of the importance of rapport to interviewing (e.g. Russano et al., 2014; Semel, 2012), the benefits of establishing and maintaining rapport throughout an interaction (see Walsh & Bull, 2012), and previous perceptions of rapport from other professionals (e.g. Goodman-Delahunty & Howes, 2016; Goodman-Delahunty et al., 2014; Redlich et al., 2014; Russano et al., 2014).

As a consequence, this research performed a combination of both inductive and deductive approaches to data coding and analysis: inductive, by means of producing codes and themes that were driven by the data, striving to give a voice to the data by ‘carving out unacknowledged pieces of narrative evidence that we select, edit, and deploy to border our arguments’ (Fine, 2002, p. 218); however, also deductive, as it is impossible for the author to be purely inductive as prior knowledge is not easily ignored (Braun & Clarke, 2012). Furthermore, prior knowledge of the subject matter under investigation can help the researcher to be sensitive to more subtle features when coding the data (Tuckett, 2005).

This research adopted the epistemological stance of Braun and Clarke’s (2006, 2012) guidance to undertaking thematic analysis by following their six phases. Phase 1 concerned the familiarisation of the data. This phase began during the transcription of the audio recorded interviews. Verbatim transcription was undertaken to reflect the participants’ interviews (Braun & Clarke, 2006) – a key process of qualitative methodology (Bird, 2005), which allowed the first author to expose themselves to the data collected. The first and second authors thoroughly familiarised themselves with the transcriptions by way of reading and rereading the data and by making notes of key phrases or discussions raised. Such notetaking is considered helpful to the process of analysis and the generation of later themes (Braun & Clarke, 2012).

Phase 2 started the systematic analysis of the data by coding standout phrases and discussions. These initial codes were either more inductive in nature, as they mirrored the language and concepts of the participants, or considered more deductive, as they invoked the authors’ prior knowledge. These initial codes acted as shorthand pithy summaries of the participants’ discussions. As initial codes are created, the first author decided whether they could be applied to the next relevant text, or whether a new code was needed (Braun & Clarke, 2012). The initial codes were tabulated within a document and reviewed to avoid repetition. This process involved merging initial codes that were similar – for example, ‘listening skills’ and ‘effective listening’ were merged to create ‘active listening’. This process was repeated until the data were entirely coded.

Phase 3 concerned the searching of themes, by merging related first-order codes to create fewer second-order codes, and finally creating themes (Hayes, 2000). A theme ‘captures something important about the data in relation to the research question, and represents some level of *patterned* response or meaning within the data set’ (Braun & Clarke, 2006, p. 82). The creation of themes is an active process, which implies themes are generated rather than discovered (Taylor & Ussher, 2001). This phase was undertaken with a mixed approach (both inductive and deductive), as the creation of themes derived from data (i.e. inductive) as well as informed by the author’s knowledge concerning rapport (i.e. deductive).

Phase 4 reviewed the potential themes, as the themes were checked against the data extracts and then in relation to the entire data set (Braun & Clarke, 2012). The coded extracts from the transcripts were labelled with the initial codes and placed under each theme. This allowed the first author to view the participants’ excerpts easily under each theme to ensure the theme represented the data. Next, the relationship between the generated themes was considered to ensure they work together in delivering an overall story of the data. Braun and Clarke (2012) note that good themes work together yet are distinctive and stand alone. The first and second authors discussed and agreed on the resulting themes (see Appendix A for thematic analysis flowcharts).

Phase 5 involved defining and naming the themes, which should be related but not overlap (Braun & Clarke, 2006). The data were interpreted with an essentialist approach, allowing the first author to explore experiences and meanings in a straightforward way. This is because an essentialist approach assumes that language reflects and enables participants to articulate meaning and experience (Potter & Wetherell, 1987). Therefore, a semantic approach to the thematic analysis was performed, in that the themes were identified within the surface meaning of the data. This process progressed from description, by summarising the semantic content and interpreting the data with regard to broader implications (Patton, 1990), and was discussed in relation to the rapport literature.

Finally, Phase 6 comprises the production of the report. In line with Braun and Clarke’s (2012) guidance, the developed themes within this research strived to build on the previous theme to tell a coherent story regarding rapport. While some qualitative research separates the discussion of the themes from the results, the present research incorporated the discussion of the literature into the analysis in order to avoid repetition. As a consequence, a ‘Results and discussion’ section was produced. An integrated approach is argued to work well when the collected data hold strong connections with existing research (Braun & Clarke, 2012). This research aimed to explore the source handlers’ perceptions and experiences of rapport during CHIS interactions in order to develop our understanding of rapport from a sample of police officers who have not previously been subjected to research.

## Results and discussion

The next section outlines the qualitative results and discusses them with regard to the rapport-based interviewing literature and policing practices of gathering HUMINT, with a particular focus on source handler interactions with CHIS. From the analysis, six themes were developed: (i) rapport is essential: ‘no rapport, no intelligence’; (ii) defining rapport within the HUMINT context; (iii) effective communication; (iv) empathy and CHIS welfare; (v) indicators of rapport: a working alliance; and (vi) training rapport. Each of these themes is discussed in turn with exemplar quotations that best demonstrate the identified themes from the participants’ responses.

### (i) Rapport is essential: ‘no rapport, no intelligence’

Participants were asked to comment on the importance of rapport in an intelligence gathering interaction with human sources of intelligence (i.e. CHIS). Rapport was perceived as a fundamental element when interacting with CHIS:

Very big, essential, if you haven’t got that rapport and you can’t build rapport with that person some people are very difficult, and even if you build the rapport it can still be very, very hard, because some people are not easy to speak to, it’s essential, without it you’re banging your head against a brick wall. (Participant 19)

Rapport is considered essential to the source handler and CHIS relationship due to the underlying objective of maintaining the relationship’s longevity. The weight placed on rapport may depend upon the situation faced by the source handler. This is eloquently outlined by Participant 4, who discusses how a source is identified, a source’s willingness to engage and the importance of joint goals as important factors in developing a rapport strategy:

If an interview is being conducted whereby the subject has identified themselves as having information of potential value to authorities, then rapport is less important than in a situation whereby the subject has been identified via other means as a person that should be approached as a potential intelligence asset. The context of this answer is that there have been numerous times whereby a person has had information that they wish to pass to the authorities, however, have no desire at all to continue with any kind of follow-up relationship. If this is the case, it should be recognised immediately, and the development of rapport should be prioritised against the importance of the information being past if this is to be a single one-off encounter. If any lasting relationship is sought; then rapport building, and maintenance could be considered as a critical part of any interview or debrief. If common ground (or the perception of common ground) and mutual respect is not established quickly, then this may jeopardise future trust or the prospect of any continued relationship. In most cases I have dealt with, there has needed to be a prompt framework of understanding between the subject and the HUMINT officer – an idea of what we both want, where our two paths coincide, what we can agree or disagree on before being able to move forward. (Participant 4)

While previous research has reported that rapport needs to be built and maintained throughout the interview (Walsh & Bull, 2012), the importance of rapport is stressed further when trying to encourage an individual to become an authorised CHIS. Further still, to then engage in such an ongoing relationship requires a level of *coordination* (Tickle-Degnen & Rosenthal, 1990) and trust in order to establish an *operational accord* (Kleinman, 2006; Tickle-Degnen, 2002). Providing a CHIS with an adequate level of trust and liking was expanded upon by one participant as a key element of rapport and potential intelligence yield:

Rapport is massive because you know it’s voluntary [being a CHIS], although they will sometimes get rewarded, it’s voluntary, you’re asking them to give up their own time, to keep it a secret from their family and from their friends, the discretion, to then want to meet you, to travel out of their area to come to see you, to do certain things before they meet you, to then go home, so you’re taking a good chunk of their life out so they’ve got to want to do that, so if they don’t like you they aren’t going to come and see you, so it’s a massive part. (Participant 20)

### (ii) Defining rapport within the HUMINT context

The definition of rapport within the context of an intelligence gathering interview was explored. From the responses, three subthemes emerged, reinforcing earlier definitions of rapport (e.g. Stanier & Nunan, 2018): (a) establishing common ground and trust; (b) reciprocity; and (c) a professional ongoing relationship.

#### (a) Establishing common ground and trust

In order to progress a relationship, a common understanding is required, which ultimately is based on trust, and an adaptive interviewing behaviour associated with enhanced cooperation from the interviewee (see Christiansen et al., 2018). Hence, establishing trust requires not simply building up the interviewee’s confidence to raise issues, but also having these addressed by the interviewer. Openness is gained through trust, by placing the interviewee at ease and by using open questions (Vallano & Schreiber Compo, 2011), thus encouraging a willingness to share information that may be actionable (i.e. intelligence). The majority of participants in the current study provided support for the process of placing the interviewee at ease, building trust and then establishing a common ground as vital to the building and maintenance of rapport:

Trying to find some common ground with the person that you’re with, so a lot of the time we’ll go into a meeting and the first part of that meeting won’t be work, it will be how are you doing? How’s the family? How are the kids? Did you watch the football? Depends on the individual or you know, did you watch the cricket? You have that knowledge because you’ve built up that understanding of the person and you are putting them at ease and you’re relaxing them and you are sort of imprinting on it that friendship that you have developed and you are also saying to them that I am not just here to get work from you, I am here to actually speak and get on with you, and for me that is the epitome of rapport, it’s that putting someone at ease and putting someone in a relaxed situation and in a trusting relationship with you, but when it comes down to the fact that you’re asking them for that information you’re getting the correct information. (Participant 16)

Prior to an interaction with a CHIS, the source handler has the opportunity to plan and prepare. Within the HUMINT context, this consists of using both open and closed sources of information to research the person of interest, as well as undertaking meetings with a source controller. However, discussions between source handlers and source controllers tend to be focused on tradecraft (e.g. how the interaction is going to take place securely and what information the source handler seeks) rather than elicitation and rapport-building techniques (Stanier & Nunan, 2018). It is therefore unsurprising that a number of participants reported that underpreparedness as being an ineffective strategy for establishing rapport, as this can lead to a limited amount of information known about the CHIS, which in turn creates fewer rapport-building *hooks* to utilise (Cooper, 2011). Hence, research can identify personal interests, lifestyle characteristics and motivations that may be utilised as rapport-building *hooks*:

I think research is important, trying to understand your candidate or customer however you want to put it, and find some themes that might resonate between the two of you and whether that is a general moral grounding on the same beliefs of wanting to improve the world or whether that is a football team or an area you have travelled to, so I think the most the important strategy for me is a bit of research and when the research fails be flexible and be guided by them. (Participant 17)

However:

Use of pre-prepared ‘script’ – very difficult to appear natural and interviewee very likely to go off script leaving you ill prepared. (Participant 1)

As Participants 1 and 17 interestingly highlight, flexibility is key to the rapport-building process, especially across numerous interactions (Christiansen et al., 2018). Hence, if the interviewer is unable to find a common ground with the interviewee, then turning the interviewee into the subject expert can (a) provide an outlet to a potentially awkward situation, (b) encourage the interviewee to talk, and (c) enhance rapport, as the interviewer will need to employ active listening to engage and show interest in the interviewee. For example:

Talking to them, asking them, and if I don’t know about something if I am with somebody [second source handler] they might be the better person to build rapport, or the other way will be if that I’m not an expert on what they want to talk about actually turning them into the expert, and actually admitting that I don’t know everything, so actually I am quite human, I don’t know much about football, I don’t know much about [football team] so tell me about it, and actually turn them into the expert which actually puts them up on a pedestal as well. (Participant 18)

#### (b) Reciprocity

CHIS understand that source handlers want to gain access to the information that they hold. Similar to Goodman-Delahunty and Howes (2016), source handlers recognised that the relationship with their CHIS cannot be one-directional, but should rather be a reciprocal relationship:

It’s the same as maintaining any good friendship that you’ve got to put the effort in, it’s got to be two way, and because any relationship has got to be reciprocal, if you don’t provide that effort you won’t get the effort back, so you’ve always got to take into account from the initial contact. (Participant 22)

Furthermore, participants stated that remembering personal details of the CHIS is an important factor for maintaining rapport, for example:

Remembering about their family, remembering about their birthdays, remembering about their holidays, remembering about important stuff in their life. (Participant 20)

This invested interest has been shown to solidify relationships (Leach, 2005; Vanderhallen et al., 2011), thus maintaining rapport:

Not showing an interest in the CHIS, they’ll pick up straight up, they’ll pick up straight away if you don’t show any interest in them, so if you say to them, if you’re sat there like, how’s the family? How’s your son? How’s your daughter? Everything alright? Good thanks, boom, move on, they will be like, really? are you interested? So you’ve got to say to them like, how’s your son? What’s he doing? Ah he’s doing this? My lads been doing that, and he’s been doing this. You interact because you pick some familiarity that you have with what they’re doing and introduce it in, because that then gives them, it’s like, ah actually he is a human being, he does think like me, things happen to him that happen to me, boom, you’ve got the relation there, there’s something they can relate to. (Participant 11)

Friendly or empathic approaches were often coupled with acts of hospitality (e.g. Goodman-Delahunty & Howes, 2016). Hence, to help build the relationship, source handlers must invest time and effort to show genuine care, understanding and empathy towards the CHIS, such as:

Doing stuff they enjoy, for instance taking them out walking, go out walking for a day, just go around the [location] just took a backpack go walking and just chat, nothing to do with the business just go and chat, just walk out, they might like motor racing take them to the races for day, you know do stuff that they like that you can just chat and get to know them a little bit, now I find that effective because the next time you go they’ll think they are investing in me, they are doing this for me, so for me an effective way is doing something not work related i.e. not trying to get intelligence out of them but just go and do something for a day. (Participant 20)

#### *(c)* A professional ongoing relationship

A commonality amongst the current participants was that rapport was considered to be the forming or building of an ongoing relationship that can be both built and lost (Walsh & Bull, 2012). Ultimately, interviewees are the source of vital information, and participants from the present study likened rapport to generating a professional friendship with the interviewee, as this may help overcome any barriers, and encourage a relationship of information exchange:

It’s for me one of the key most important things, I think from that initial either handshake in the front office or the phone call you make to get them into a police station, however it is you’re going to do it, just actually speaking to somebody professionally, properly, politely, all those basic things which sometimes get taken for granted, that is the start of the rapport. (Participant 14)

Participants discussed rapport as critical in providing the interviewee with the confidence to open up, positively challenge the interviewer, declare concerns, to ensure that the CHIS does not put themselves at risk and to enhance a professional working alliance (Tickle-Degnen, 2002; Vanderhallen et al., 2011). One participant further acknowledged the importance of establishing rapport that was built on a professional foundation:

It’s a fine balance I believe, it’s a fine balance between being friendly with somebody and that person believing that you’re their friend, but you have to have that professional part of you where your, when I’m in that world I’ll be your friend but as soon as I step out of that world I am the professional that I need to be, but when I come and meet you I’m your friend, they have to believe that, because when you’re their friend, they will tell you all kinds of things, if they see you as the authorities what they tell you might be very clipped. (Participant 11)

Moreover, underpinning the relationship requires a professional boundary. Though an informal and friendly approach is encouraged to open up the interviewee, if the relationship loses its professional foundation a CHIS may end up at risk:

I’ve got a source at the moment who I’ve had to take them to task and say, look you need to sort of switch on, because he sees me as his mate and there’s the issues, some of the stuff that started happening was woah hang on a minute, yeah we are friends however this is a professional relationship, don’t cross the boundary because then your safety then gets put at risk, and we don’t want that to happen, you’ve got to identify that. (Participant 11)

Although many participants acknowledged that an element of friendship was important to the relationship, ultimately, a level of continued professionalism demands a level of reciprocity, it can keep the interaction focused, and most importantly, it ensures the CHIS’ welfare is in check (Billingsley, 2003):

You have to get that information and also how many people know what the source knows, so if the source is saying to you Mr X and Mr Y are doing this, this and this, on this day, you need to then be saying, right ok well how many people know that information? Well only I know that information, well then what can we do with that information? We’ve got that information but if we then leak that information out our source gets burnt, so then we have to parallel that information. (Participant 11)

Participants frequently associated the source handler and CHIS relationship with the notion of *operational accord* (Kleinman, 2006). This is because an appreciation of the interviewee’s concerns and intentions together with the desired outcomes of the interaction are considered important elements of rapport (Evans et al., 2010):

Rapport is ongoing. When you have run a CHIS for a long time, it is about not becoming overly personal with them but continuing to be professional and to a certain extent a friend or support to the CHIS from time to time. It is worth reviewing your relationship with your CHIS from time to time in a reflective way. It is worth debriefing meetings with a controller or co-handler. It is also worth using the services of [operational partners] which can offer invaluable insight into aspects of your CHIS. (Participant 5)

### (iii) Effective communication

The elicitation of timely, detailed and reliable intelligence is vital for subsequent investigative decision-making (James, 2013), which subsequently influences the outcomes of proactive and reactive criminal investigations (Chappell, 2015; James et al., 2016). Thus, to maximise the elicitation process, it is important that the Source Handler has adequate knowledge of the intelligence requirement. This is because, even if all the elicitation techniques are maximised, if the questions themselves do not elicit relevant information, then the overall interaction is sub-optimal. One participant noted that the aims and objectives of the interview are just as important as rapport, as they ultimately work hand in hand:

So I would say, knowledge of why you’re there, knowledge of your subject, knowledge of what you’re after, your aims, your objectives sits right next to rapport, because you could have all the rapport in the world but how do you steer the conversation if you don’t know what you are there for, secondly if you know exactly why you’re there but you have no rapport with the individual the conversation doesn’t take place, so they’ve got to be equal. (Participant 21)

The PEACE model of investigative interviewing could be successfully applied to the intelligence interview (e.g., Stanier & Nunan, 2018), especially when such importance is placed upon the planning and preparation of an interview, as well as a need for rapport development and maintenance throughout (Clarke & Milne, 2001, 2016; Walsh & Bull, 2012).

Interviews that possess overly officious interactions, and therefore lack a working alliance have been argued to impede rapport (Milne & Bull, 1999), and this especially applies within an intelligence gathering context whereby the interaction will most likely encourage cooperation through informal practices, rather than conducting a suspect-like interview with a CHIS:

Just coming straight to the point in what you’re after, no pleasantries, no rapport building, just literally, thanks for coming in, this is what I want, have you got it, yes or no? Okay leave, I think that is a way to close your subject down and not get much from the relationship. (Participant 14)

However, the formality can depend upon the CHIS, and it is part of the Source Handler’s role to understand what works. Participants discussed considerations such as what time and location is convenient to the CHIS, and where they would feel most relaxed.

Half of the participants stated that effective communication skills and style were beneficial to establishing rapport. Incorporated within effective communication is effective listening, a skill considered vital to a successful interview (Milne & Bull, 1999). In an attempt to establish a common ground with the interviewee, effective listening can provide the interviewer with information relating to the interviewee’s interests:

Very often if you listen for long enough you find what people want to talk about rather than going in with your own preconception, research in advance, brilliant, but then listen and let’s find out what that individual wants to talk about. (Participant 17)

Moreover, participants stated that effective verbal communication with a CHIS is through soft intelligence questions such as indirect questioning, supported by the following:

Paraphrasing, effective interaction between handlers, tone, and effective use of pauses. (Participant 6)

With regard to nonverbal rapport, effective observation of the CHIS’ nonverbals, as well as effective use of nonverbal behaviour on the interviewer’s part was considered important by participants in the current study and by previous research (Goodman-Delahunty et al., 2014; Russano et al., 2014). Examples included mirroring and:

Good use of eye contact, good use of NVC’s [nonverbal cues], and a handshake to establish appropriate personal contact. (Participant 5)

The implementation of effective nonverbal techniques (e.g., mirroring behaviour and displaying understanding via empathy) has also been perceived by other interviewers as effective rapport building techniques (Abbe & Brandon, 2013; Vallano et al., 2015).

### (iv) Empathy and CHIS Welfare

It is important to note that RIPA legally mandates the security and welfare of a CHIS to be monitored. Nonetheless, a demonstration of empathy towards the CHIS’ circumstances and welfare was perceived to be an effective rapport-building strategy. This may be demonstrated by displaying humanity and care towards the CHIS by trying to identify their worries and concerns (Abbe & Brandon, 2013):

On some occasions acting on what they’re saying, so even if it’s got nothing to do with the reasons why you’re there, it’s important to them so it’s something that should be given some sort of attention, I suppose examples would be if there is an event going on in their life which has got nothing to do with what I’m there for, I’ll perhaps put a welfare call in, in between my sort of process, and just purely to talk about that incident in their life, whether it be a children’s football match or something just to show I was listening to what they said and in actual fact I’m paying an interest and attention, and then I won’t ask anything from them on that call. (Participant 14)

A third of the participants reported that a lack of empathy has a negative influence on rapport, by not addressing welfare concerns (i.e. a frustration in delays in reward payments or concerns regarding the taskings). Moreover, as the role of a source handler is to elicit information that is often highly personal, a lack of empathy has been shown to be damaging towards an existing relationship (Risan, Binder, & Milne, 2016) and a barrier against effective rapport building.

Throughout the process of establishing trust and common understanding, an interviewer’s empathy was considered important to the process of rapport. Empathy was frequently found to be well received by interviewees when sharing highly personal information, a finding entrenched in therapeutic settings (e.g. Leach, 2005; Miller & Rollnick, 1992). Empathy can take many forms, and cover a number of a CHIS’ circumstances:

Social, economic, religious consideration of the source, taking account of the sources mental state. Having a consideration for any medical needs, alternative meeting arrangements and locations. Basing the debrief initially around rapport building, researching the above factors so that common interests/hobbies etc. can be discussed. (Participant 9)

As noted, the CHIS’ welfare was perceived as highly important, especially with regard to maintaining rapport. Participants stated that basic humanity, being supportive and demonstrating an understanding of the CHIS’ circumstances all form part of providing welfare attention. Additionally, source handlers providing easy, regular and convenient contact was perceived to be key to reinforcing the notion of taking an interest:

Regular communication that is not interrupting in any way in time or place. (Participant 23)

### (v) Indicators of rapport: a working alliance

Across the data, participants discussed an array of indicators that they perceived as demonstrating rapport. Rapport was discussed by some participants with regard to its influence on intelligence yield. Previous research suggests that rapport-based interviewing supports information disclosure across numerous interviewing contexts (Alison et al., 2013; Goodman-Delahunty et al., 2014), as well as being perceived to be the most effective interviewing approach (Redlich et al., 2014). Participants’ perceptions from the current study were found to be aligned with such evidence. Rapport was considered important to the CHIS’ openness, thus, impacting on not only intelligence quantity but also quality:

A relaxed CHIS is going to give you the best intel product, if they’re at ease and there is no issues and they are wanting to tell you that information because of the relationship and rapport you’ve built up with them, then you’re going to get the best product from them, and the most untainted product, because it’s all about encouraging someone to openly speak to you, and the best way to do that is to get on with someone, as it is in all walks of life, if you get on with someone you’re more likely to speak to them in a nice open way and just talk . . . it’s that open bit that’s the key bit, because if they’re closed you’re not going to get the full picture. (Participant 16)

Furthermore, participants discussed the CHIS’ work ethic towards a task set by them as a way of understanding whether rapport was present. In line with the development of a working alliance (Vanderhallen et al., 2011), participants equated the CHIS’ work ethic to having rapport with that individual. This was demonstrated by a:

Willingness of CHIS to go the ‘extra mile’ to satisfy a tasking (Lawfully!!). (Participant 2)

as well as a:

General upbeat positive attitude. The source themselves asking for opportunities for development that the handling team may have missed. Having a genuine interest in the subject matter. Regular positive outcomes from tasking opportunities. (Participant 9)

The tasking outcome was also perceived to be an important indicator of rapport, whereby participants alluded to both detailed and reliable intelligence. If the use of rapport has been shown to assist elicitation in a number of interviewing contexts (e.g. Alison et al., 2013; Goodman-Delahunty et al., 2014; Redlich et al., 2014; Vallano et al., 2015), then using the tasking outcome or intelligence yield (e.g. ORBIT; Alison et al., 2013) may be one way of demonstrating that rapport is present.

From a nonverbal perspective, participants noted that observing, and to some extent sensing, the CHIS’ relaxed body language (e.g. take their coat off, smiling or mirroring the source handler’s behaviour) was one way of knowing rapport had been achieved. This resonates with being comfortable in the interaction and is exemplified by the following participant’s response:

Body language, laughter, smiling, eye contact, if they do relax, if they do take a drink off you, you know it’s just getting that whole sort of, it’s hard to sort of vocalise really, it’s just its understanding, looking at the person, yes, they’re relaxed, it’s like an intuition really, I know it sounds probably silly but it’s pretty intuitive this game. (Participant 13)

With regard to the verbal aspect of an interaction, what is divulged by the CHIS and how they share that information was perceived to be an important indicator of rapport. As with personal relationships, the amount of personal information shared can heavily depend upon the existing relationship with the person receiving that information. A lack of rapport, as a result of maladaptive behaviours (e.g. judgemental, unfriendly or distrustful; Alison & Alison, 2017) can quickly generate negative perceptions of the interviewer and thus close down the interviewee’s willingness to share meaningful information (Russano et al., 2014; Semel, 2012). Hence, when an interviewee begins to share personal information, this may be a strong indicator of rapport, and that the interviewee feels the relationship is at an appropriate level to divulge such information:

How they’re speaking to you, I think if they’re openly discussing things with you I think that’s a big one, some people might hold back in the first one or two meetings but as the relationship progresses they start telling you more about their personal circumstances, I think also you start seeing a personality of that person, so rather than just being sort of straight faced, they might start laughing and joking and throwing a little bit of themselves into it, so yeah easy to speak to. (Participant 19)

A relaxed environment, through both verbal and nonverbal techniques, not only has been found to influence an individual to share information (i.e. quantity) but can also positively impact on the quality of memory recall (Vallano et al., 2015). Moreover, rapport-based interviewing may encourage multiple retrieval attempts (Memon et al., 1997), which, supported by the use of open-ended questions, should maximise the elicitation of intelligence (Vallano & Schreiber Compo, 2011).

### (vi) Training rapport

A number of participants (*n* = 7) perceived that rapport could not be something that a person can be trained to develop, suggesting that rapport appears feigned if a person does not possess an innate ability to build rapport, for example:

I think you can assess how comfortable somebody is at building rapport and certainly within [previous training] there are elements of that course that focus on that, so they’ll take you into a public house environment and tell you to strike up a conversation with two different people in there and extract x number of pieces of information from them, so you can assess how comfortable somebody is as doing that, but if somebody is not comfortable at doing it I am not convinced you train them to be comfortable. (Participant 17)

However, the majority of participants (*n* = 17) believed that training can help people to build rapport. Participants noted that for rapport training, individuals require an existing natural basis of interpersonal skills, which in turn can be developed through training. Participants perceived interpersonal skills to involve elements of verbal and nonverbal communication techniques, adequate self-awareness, being personable, and genuine empathy (Redlich et al., 2014; Risan et al., 2016). One participant compared training rapport to interview training:

Can you train someone to interview? Yeah you can, you can teach them a legislative framework, are they going to be naturally good at it? Maybe yes, maybe no, natural communicators are people who can naturally interview, an interview is just a conversation with some legal framework, rapport building, if you’re not the sort of person who walks in, hi how you doing? Big smile, bit of eye contact, bit of confidence, then you’re probably never going to do it, it’s almost like a little bit false and stuttery, you can become better at it, you know there are some good skills and tricks you can teach people, but you know things naturally we do, when I am talking to you we do nod, we smile, we want to send out those receptors that you’re going in the right direction, almost here now if I was saying something completely batty, you don’t agree with, you’re not there shaking your head tutting, because I will dry up very quickly . . . you know it doesn’t matter if you agree with it, what matters is they’re talking, so I think you can train it to a point, I think there’s some natural skills, some people are naturally more gregarious, we look at how people are recruited in radicalisation, you know they are naturally gregarious, if you ask someone to sit down and say why were you radicalised? What was that person like? They were engaging, they were gregarious, I had confidence in them, what do you want from your handler? Oh, I want them to be gregarious, have confidence in them, so there’s quite similar skills those people with manipulation skills. (Participant 24)

Participants referred to training rapport by highlighting techniques that assist with rapport building and its maintenance, which included training on social psychology, communication and persuasion:

I’ve been taught it by a lecture or a training day, material about reciprocity, liking, authority, scarcity, social proof, commitment and consistency, a body of work by Robert Calidini about sales techniques, how to build the rapport and relationship to sell them a product, all that stuff applies within CHIS handling, so I suppose if you teach that you can teach rapport building. (Participant 22)

It is likely that source handlers already implicitly use motivational interviewing skills; however, training that incorporates motivational interviewing may reinforce effective interviewers to become more aware of the skills they are using to build rapport (Alison et al., 2015). Further still, it is important that source handlers are aware of how maladaptive behaviours may be detrimental to rapport (Alison et al., 2014) and ultimately intelligence collection (Alison & Alison, 2017).

By building upon the natural communication skills that already exist within the source handler, the development of effective communication is the foundation of both establishing and maintaining rapport. Additionally, one participant highlighted that:

I think they can train the handlers to identify how they can get the hooks into the person. (Participant 11)

Training source handlers to identify the *hooks* of a CHIS refers to quickly understanding the CHIS’ motivations (Billingsley, 2001) and establishing a common ground, and using this to influence rapport, thus in turn assisting with elicitation. Perceived to be trainable, this effective technique could significantly impact upon the outcome of an interaction, especially in relation to ideological *hooks*, which have shown to be influential motivators for CHIS (e.g. Cooper, 2011).

Finally, participants perceived that learning from good examples and scenario-based training were effective ways of training rapport. Exposure to various settings was considered highly beneficial to rapport development, such as training in a safe environment, learning from previous life experiences and learning from other colleagues:

Seeing how the other guys are building rapport and how they’re engaging with an individual, subtly you go through that training in as much as, ah so you had somebody that does that, and they go oh I particularly like that bit, the comment that they made, the rapport that they’ve established by touching on that particular subject. . . . but I know that for me it more than likely wouldn’t sound right but I can do the same if I make it more personal to myself, so it’s making it more comfortable when I say it, so I would say working with other people, learning it on the job and then adapting it to your own personal benefit. (Participant 21)

Overall, it was found that source handlers can be made aware of techniques that can be employed to assist with rapport (i.e. mirroring, informal introductions, politeness) and through practice (e.g. various scenarios). They perceived that training rapport can help source handlers identify strategies (i.e. *hooks*, Cooper, 2011) that work for them to build relationships with CHIS (Billingsley, 2001). With rapport considered essential to the outcome of an intelligence gathering interaction (e.g. recruitment, intelligence yield, maintaining the relationship, persuading someone to meet again; Alison et al., 2013; Goodman-Delahunty et al., 2014; Russano et al., 2014; Stanier & Nunan, 2018), this should reinforce rapport as a vital element of source handler training, which is currently lacking from national source handler training courses.

## Limitations and future directions

The present research achieved privileged access to a unique sample of police source handlers who work within counter-terrorism dedicated source units: professionals who have not previously been researched. While it is acknowledged that the sample only comprised 24 participants, a number of counter-terrorism hubs across England and Wales were represented, and all counter-terrorism source handlers are trained to the same national standard. Counter-terrorism source handling is a specialist policing role, which, as a subsection of police officers are relatively small in numbers. The present self-reported data provided a representative insight into the participants’ perceptions and experiences of rapport with CHIS, allowing an element of transferability of the results. However, rapport is a dyadic relationship, and the present research has only addressed the perceptions of one side (i.e. the source handler). Future research may wish to address this by exploring the perceptions and experiences of intelligence sources (subject to appropriate vetting and access). It is acknowledged that research based upon self-reported data is susceptible to socially desirable answers and inaccurate memories of past events (Robson & McCartan, 2016). Hence, the structured interview protocol consisted of open-ended questions, which did not prompt participants for answers. Since reported perceptions and experiences may differ to actual behaviour, the next phase of this programme of research coded rapport in real-life audio recorded source handler interactions.

## Conclusion

This research is believed to be the first of its kind in exploring the perceptions and experiences of police source handlers from England and Wales counter-terrorism dedicated source units. It was identified that rapport was perceived to be essential to the collection of HUMINT, with participants stressing the importance of building and maintaining rapport. Effective communication, establishing common ground and trust, reciprocity and a concern for welfare were considered key to rapport. The majority of participants believed rapport could be trained to some degree. While rapport was not viewed exclusively as a natural skill, participants perceived that some natural attributes are required to build rapport, with those natural attributes being refined and developed through training and experience.

Rapport-based interviewing has been shown to be effective in a range of contexts (e.g. Alison et al., 2013; Christiansen et al., 2018; Goodman-Delahunty et al., 2014; Redlich et al., 2014; Russano et al., 2014; Semel, 2012), and the present research adds to that evidence-base. The fact that a sample of specialist police officers, who have not previously been the subject of research, perceive and experience rapport similarly to other law enforcement professionals should be considered a strength that advances our understanding of rapport, rather than a limitation. An appreciation of the perceptions and experiences of HUMINT practitioners advances the academic literature, highlights areas for future research and may in turn inform practice.

This research therefore concludes that rapport should be considered fundamental to the source handler and CHIS relationship, due to its perceived impact on maximising intelligence elicitation. Taken together, the training methods and rapport behaviours discussed by source handlers in light of previous research should be implemented into the national source handler training course. Not only should source handlers be made aware of adaptive behaviours of rapport that are beneficial, it is vital that they are also aware of how maladaptive behaviours may be detrimental to rapport (Alison et al., 2014) and ultimately intelligence collection (Alison & Alison, 2017).

## Appendix 1 Appendix A: Thematic analysis

Note: This flowchart continues onto the next page, therefore the theme is repeated.
